# Quinoa Fibre Isolated By Wet Milling as a New Ingredient for Food Enrichment: Nutritional Value and Technological Properties

**DOI:** 10.1007/s11130-025-01409-5

**Published:** 2025-10-18

**Authors:** A. Alonso-Álvarez, C. M. Haros

**Affiliations:** Cereal Group, Instituto de Agroquímica y Tecnología de Alimentos (IATA-CSIC), Av. Agustín Escardino 7, Parque Científico, Paterna, Valencia, Spain

**Keywords:** Chenopodium quinoa, Quinoa fibre, Wet milling, Nutritional value, Food ingredient, Mineral bioavailability

## Abstract

Wet-milled quinoa fibres show strong potential as ingredients in nutritionally enhanced food products, offering a valuable strategy to address dietary fibre deficiencies. In this study, white, red, and black Royal Bolivian quinoa were wet-milled to produce fibre-rich fractions and assess their nutritional and techno-functional properties. All fibre-rich fraction samples showed a marked increase in dietary fibre content compared to whole grain flours—about six-fold in white quinoa and eight-fold in red and black. Insoluble fibre remained predominant, increasing by approximately eight to ten times, with the highest increase observed in red quinoa, while soluble fibre increased by two to three times, likely due to partial solubilisation during processing, reaching its maximum in black quinoa. A 5 g serving of these fractions could supply 10–14% of the WHO’s daily fibre recommendation (25 g), compared to only 2% from whole quinoa flour. Fibre recovery was highest in black quinoa, followed by white and red. Calcium and iron contents increased in the isolated fibres, while zinc decreased, especially in red and black varieties. Phytate content, a mineral absorption inhibitor, was significantly reduced (by 45–85%), which can enhance mineral bioavailability. Notably, the black variety showed favourable phytate-to-mineral molar ratios, indicating no inhibition of calcium or zinc absorption. Functionally, red quinoa fibre exhibited better water and oil retention and higher swelling capacity, properties desirable in bakery and meat product formulations, while black quinoa fibre stood out for its high total fibre content. Overall, wet-milled quinoa fibres offer promising potential as functional, fibre-rich ingredients for healthier food formulations.

**Supplementary Information:**

The online version contains supplementary material available at 10.1007/s11130-025-01409-5.

## Introduction

**Fig. 1:**
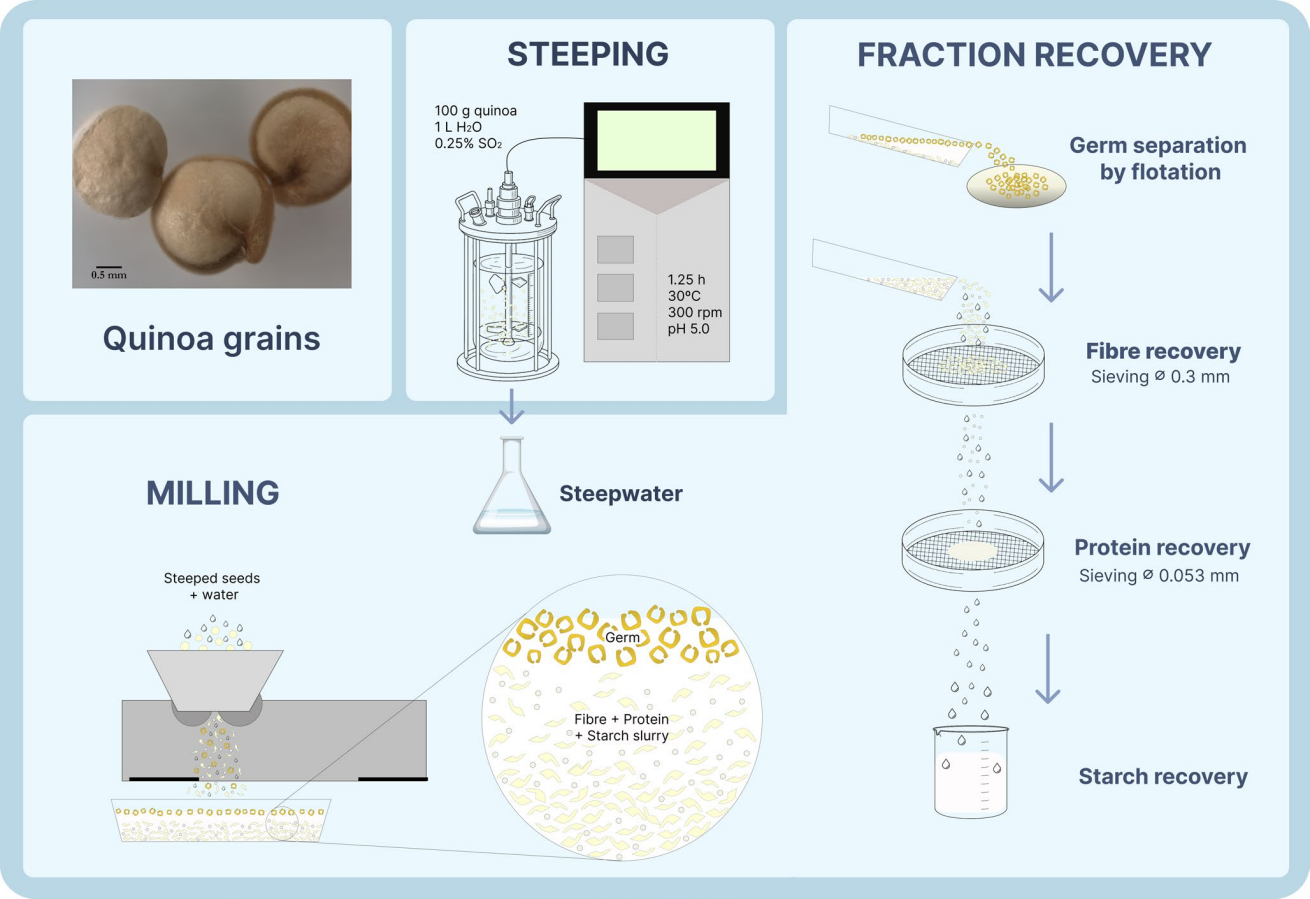
Lab-scale quinoa wet milling

Quinoa (*Chenopodium quinoa Willd.)*, classified as a pseudocereal, is available in a range of seed colours, including white, yellow, red, purple, and black. Although taxonomically distinct from cereals, pseudocereals like quinoa are often grouped with them due to their comparable starch content and similar seed composition. Historically, quinoa was extensively cultivated and consumed by pre-Columbian civilisations across the Andean regions of South America [1]. A distinguishing feature of quinoa is its pigmentation, which not only defines the visual appeal of the grain but also reflects its phytochemical profile. Darker varieties, such us red and black quinoa, contain higher levels of bioactive compounds (phenolic acids, flavonoids, and betalains) compared to white quinoa [2]. These antioxidants, mainly in the outer layers of the grain, contribute to the antioxidant capacity and potential health benefits of coloured quinoa varieties. In addition to its antioxidant properties, quinoa is particularly valued for its high-quality protein content, offering a well-balanced profile of essential amino acids comparable to that of animal proteins, with elevated levels of lysine, histidine, and methionine (amino acids that are typically limited in cereals and legumes). Quinoa also provide a wide range of essential vitamins and minerals [3]. The absence of prolamins makes quinoa especially suitable for the formulation of gluten-free food products [4]. Furthermore, quinoa contains a lipid content approximately two to three times greater than that of conventional cereals, with over 75% comprising unsaturated fatty acids, particularly oleic and linoleic acids [3]. Quinoa is also an excellent source of dietary fibre, which contributes to its functional properties [1]. Techno-functional properties of dietary fibres, such as water-holding capacity (WHC), oil-holding capacity (OHC) and swelling capacity (SC), are key parameters to evaluate their potential use as food ingredients. WHC reflects the ability of fibre to retain water within its structure, influencing texture, viscosity, and product stability [5, 6]. Oil-holding capacity (OHC) refers to the capacity of fibre matrices to entrap oil, which is mainly related to surface properties and porosity, and is of interest for developing products with lower energy density or improved lipid binding [7, 8]. SC indicates the volume expansion of fibre upon hydration, contributing to product freshness, stability, satiety, and faecal bulk [9]. Together, these properties provide insight into the functional performance of dietary fibres in various food applications. A higher proportion of quinoa fibre is insoluble, which supports gastrointestinal health and enhances satiety, while also playing a role in modulating postprandial glycaemic response [10]. The soluble fraction has been associated with improvements in lipid metabolism and blood glucose regulation [11]. In comparison with traditional cereals, quinoa often presents a higher total fibre content (3.8–14.1 g/100 g, dry matter), reinforcing its potential in the development of functional and fibre-enriched and gluten free-products, especially in the context of globally insufficient dietary fibre intake (< 25 g/day) [1, 12]. However, an adequate intake of dietary fibre has a positive effect on nutrient bioavailability due to its physical properties within the gastrointestinal tract, including its cation-exchange capacity, water-holding ability, viscosity, and fermentability. These attributes could affect the absorption of fatty acids by forming viscous gels in the small intestine, thereby modulating nutrient interaction and transit [13]. In the colon, microbial fermentation of dietary fibre generates short-chain fatty acids (SCFAs); primarily acetate, propionate, and butyrate; whose profiles depend on fibre type [14]. The acidic environment resulting from SCFA production enhances mineral solubility, promoting improved calcium absorption [15]. To improve quinoa’s functional profile and expand its application, the adaptation of wet milling, commonly used in cereal processing such as maize to pseudocereals like quinoa presents a promising strategy for the fractionation and recovery of key grain components, including starch, fibre, germ, and proteins (Fig. 1). This approach not only allows for targeted use of individual fractions, but fibre isolated via wet milling has also been reported greater yields with higher purity compared to traditional dry milling [2]. An additional benefit of this method is the potential reduction of phytates, antinutrient commonly found in pseudocereals, cereals, oilseeds or legumes [16]. Phytates (*myo*-inositol hexakisphosphate) can strongly chelate essential minerals such as iron, zinc, and calcium, thereby limiting their bioavailability. However, the steeping step in wet milling, which involves hydration and moderate temperatures, may contribute to a reduction in the phytate content of the isolated fractions.

This study aimed to obtain fibre-rich fractions from white, red, and black quinoa through a wet milling process, as potential ingredients with improved mineral bioavailability. These fibre fractions were characterised and compared in terms of their physicochemical composition (proximate composition, minerals, phytic acid, colour parameters and particle size distribution) relative to their source grains. Furthermore, their technological (water holding capacity, oil holding capacity and swelling capacity) and nutritional potential was evaluated for application in food product development.

## Materials and Methods

### Raw Materials

White, red and black Royal quinoa grains (*Chenopodium quinoa* Willd.) produced in Bolivia were sourced from the Organic Quinoa Real^®^ commercial brand. The grains were milled separately using a household grinder (Aromatic, Taurus, Oliana, Spain) to produce wholemeal flour, which was stored at 4 °C.

### Quinoa Fibre Isolation

The fibre-rich fraction of each quinoa type was individually isolated through laboratory-scale wet milling (Fig. 1), applying the steeping conditions optimised by Ballester-Sánchez et al. [17] to maximise fibre yield. Their factorial design assessed the effects of steeping temperature and time on the separation of quinoa fractions, identifying 1.25 h at 30 °C as optimal for fibre recovery. Quinoa seeds (100 g) were steeped in 1 L of sodium bisulphite solution (0.25% SO_2_, pH adjusted to 5.0 with lactic acid) under controlled agitation (300 rpm) and temperature in a laboratory fermenter (Biostat Bplus, Sartorius, Spain). After steeping, the grains were milled using a laboratory-scale plate mill (Corona, Lambers & Cia, Colombia). The germ fraction was removed by flotation in water and rinsed with milli-Q water (1 L) to eliminate residual starch. The remaining slurry was passed through a 300 μm sieve to collect the hulls, corresponding to the fibre-rich fraction. This material was washed with milli-Q water, dried overnight at 40 °C in a forced convection oven and stored at 4 °C in vacuum-sealed polyethylene bags. Fibre yield was expressed as the dry weight of the isolated fibre-rich fraction relative to the initial dry weight of the quinoa seeds, and reported as a percentage.

### Supplementary Methods

Detailed procedures for the chemical proximal composition, phytate determination, mineral analysis, physical and technofunctional properties of fibre fractions, and statistical analysis used are provided in the supplementary material.

## Results and Discussion

### Proximate Composition

The proximate composition of fibre quinoa fractions (white, red and black) and their respective whole grains is presented in Table 1. Starch, the major component of quinoa grain, was notably reduced in the fibre fractions obtained via wet milling, with red and black varieties showing the lowest residual levels. Concurrently, dietary fibre content increased significantly across all quinoa varieties, approximately six-fold in white variety, and eight-fold in red and black varieties compared to their original grains. The resulting fibre-rich fractions exhibited high purity, with dietary fibre representing the main component: approximately 57% in white, 74% in red, and 76% in black quinoa. These values are consistent with the 68% purity reported by Ballester-Sánchez et al. for red quinoa fibre obtained via wet milling, and surpass the purities typically achieved through dry milling, highlighting the efficiency of wet processing in isolating highly pure fibre fractions. Quinoa is generally richer in insoluble than soluble dietary fibre, as observed in all samples analysed. Nutritionally, insoluble fibre contributes to improved gut motility, stool bulk, and reduced intestinal transit time. In the fibre fractions, insoluble fibre was enriched by approximately 8–10 times, while soluble fibre increased only 2–3 times, likely due to partial solubilisation and loss into the steeping water during wet milling. This differential enrichment resulted in notably higher insoluble-to-soluble fibre ratios in the final fractions, reflecting a selective concentration of the insoluble component. The total dietary fibre (TDF) content of quinoa fibres (Table 1), particularly those derived from red and black quinoa, was remarkably high, comparable to or even exceed those reported for commonly recognised high-fibre sources such as arame algae (74.6 g/100 g dry matter, d.m.), lemon peel (62.7 g/100 g d.m.), and grape peel (55 g/100 g d.m.), as outlined by Elleuch et al. [18]. In contrast, traditional cereal by-products such as wheat bran and rice bran showed considerably lower TDF values, at 44.5 g/100 g and 27 g/100 g, respectively [18]. Regarding nutritional relevance, a 5 g serving of these fibre-rich ingredients would contribute between 10 and 14% of the adequate intake of 25 g/day recommended by World Health Organization, compared to only 2% from the original whole quinoa flours, highlighting their potential as functional dietary fibre sources. Fibre extraction yields differed significantly among quinoa varieties studied, with black quinoa exhibiting the highest recovery (20.6% d.m.), followed by white (17.1% d.m.) and red (14.6% d.m.). These differences are closely linked to the distinct structural characteristics of each grain, including pericarp thickness, cell wall integrity, and the distribution and composition of outer layers, which can affect the separation efficiency during wet milling. In particular, varieties with a more open structure or higher hydration capacity tend to soften more effectively during steeping, facilitating the disaggregation of fibrous tissues [16]. Furthermore, the phenolic profile of each variety may influence extraction, as phenolic compounds, especially when bound to cell wall polymers, can contribute to cross-linking and structural rigidity, thus reducing fibre recovery [16]. It is also important to note that the wet milling is a complex, multi-stage process, and both yield and quality of the extracted fibre are strongly influenced by steeping conditions, including pH, temperature, steeping time, and the presence of SO_2_ [19], which modulate the softening of tissues and solubilisation of non-fibrous components. Under comparable conditions, quinoa generally yielded higher fibre recovery under wet milling compared to other cereals; for instance, maize has shown lower yields (15.4% d.m.), likely due to its compact pericarp and lower structural polysaccharide content [16, 20]. These differences highlight the role of grain structure and composition in extraction efficiency, reinforcing quinoa’s potential for fibre-rich ingredient production. The remaining macronutrients - proteins, lipids, and ash – showed varying responses to the wet milling process. Notably, protein content increased significantly in the fibre fractions, particularly in red quinoa fibre, which exhibited the highest value compared to its original grain. A moderate increase was also observed in white and black quinoa fibres. This enrichment may be attribute to the partial removal of starch and concentration of protein-rich embryo tissues, which tend to co-separate with fibre during wet milling. In contrast, lipid levels remained relatively stable across samples, with no significant reduction in the fibre fractions. An exception was observed in the white quinoa fibre, which showed a slight but significant increase in lipid content, possibly due to retention of germ residues, where oils are concentrated. Ash content was also largely preserved, although white quinoa fibre presented a notably lower value, which could be associated with the loss of mineral-rich perisperm and seed coat components during steeping or separation. Overall, quinoa’s nutritional richness (notably its content of high-quality protein, unsaturated lipids, and essential minerals) is primarily located in the germ and outer layers of the seeds [1]. These anatomical characteristics help to explain the nutrient profile observed in the fibre fractions, as components retained or lost during wet milling are closely linked to their spatial distribution within the grain.

**Table 1:** Proximate composition of Quinoa flours and fibres

Parameter^a^	Units	Quinoa flours			Quinoa fibres		
		White	Red	Black	White	Red	Black
Moisture^c^	g/100 g	11.3 ± 0.9a	10.1 ± 0.1a	10.6 ± 0.4a	10.9 ± 0.3a	10.6 ± 0.0a	10.5 ± 0.0a
Starch^b^	g/100 g	65 ± 4d	57 ± 2c	60 ± 1 cd	27 ± 2b	11 ± 1a	14 ± 2a
Proteins^b^	g/100 g	13.5 ± 0.2a	13.7 ± 0.2a	14.0 ± 0.1a	15.2 ± 0.1c	19.4 ± 0.3d	14.7 ± 0.1b
Lipids^b^	g/100 g	5.3 ± 0.1a	5.7 ± 0.3a	5.0 ± 0.5a	7.7 ± 1.4b	4.5 ± 0.8a	4.1 ± 0.2a
Ash^b^	g/100 g	2.2 ± 0.1b	2.3 ± 0.0b	2.3 ± 0.2b	1.3 ± 0.1a	2.3 ± 0.1b	2.2 ± 0.1b
TDF^b^	g/100 g	8.7 ± 0.1a	9.0 ± 0.1a	9.0 ± 1.7a	56.7 ± 0.5b	74.1 ± 0.4c	75.9 ± 3.0c
IDF^b^	g/100 g	6.1 ± 0.3a	6.9 ± 0.7a	7.8 ± 1.4a	49.7 ± 0.4b	69.0 ± 0.4c	72.6 ± 3.3d
SDF^b^	g/100 g	2.6 ± 0.2bc	2.2 ± 0.7ab	1.2 ± 0.3a	7.1 ± 0.2e	5.2 ± 0.4d	3.3 ± 0.5c
IDF: SDF		2:1	3:1	7:1	7:1	13:1	13:1
AI Contribution	%	2	2	2	10	13	14

^a^Values are expressed as mean ± standard deviation (*n* ≥ 3). Values followed by the same letter in the same line are not significantly different at 95% confidence level. ^b^ Dry matter. ^c^ Wet basis. Abbreviations: TDF: Total Dietary Fibre; IDF: Insoluble Dietary Fibre; SDF: Soluble Dietary Fibre; AI: Adequate Intake. Values refer to dietary fibre for adults, calculated based on an intake of 5 g

### Mineral Content and its Contribution To the Dietary Reference Intakes (DRIs)

The mineral content of quinoa fibres and flours is shown in Table 2. Calcium, iron, and zinc levels obtained for quinoa flours were comparable to those reported by other authors (Ca: 32.7-148.7; Fe: 4.7–13.2; Zn: 1.8-5.0 mg/100 g) [1, 16]. Calcium and iron were concentrated in the fibre fractions across all varieties, particularly for the white and red quinoa samples. In these cases, the fibres presented significantly greater amounts of calcium and iron than their respective flours, confirming a clear enrichment in the fibrous portions of the grain. In contrast, zinc content was generally lower in the fibres than in the flours, especially in red and black quinoa. This reduction was less pronounced in the white variety, where no significant differences were found between flour and fibre. This pattern may be explained by observations in buckwheat and wheat, where zinc is primarily localized in the embryo and aleurone layer, rather than in the fibre fraction [28, 29] These differences highlight that quinoa fibre fractions could serve not only as a source of dietary fibre, but also as a valuable ingredient in the development of foods with enhanced calcium and iron content, mainly in formulations targeting nutritional fortification.

**Table 2:** Mineral contribution, phytate content and estimated bioavailability of Quinoa flours and fibres^a^

Parameter			RDA or PRI male/female	Quinoa flours			Quinoa fibres		
				White	Red	Black	White	Red	Black
Calcium^b^, mg/100 g			–	31 ± 1a	47 ± 3ab	71 ± 5 cd	68 ± 9 cd	59 ± 8bc	82 ± 16d
Iron^b^, mg/100 g			–	4.3 ± 0.2a	5.2 ± 0.1ab	6.3 ± 0.4bc	9.1 ± 0.4e	7.6 ± 1.5d	7.6 ± 1.3 cd
Zinc^b^, mg/100 g			–	3.1 ± 0.3b	3.2 ± 0.4b	3.2 ± 0.3b	2.9 ± 0.2b	2.4 ± 0.5a	2.2 ± 0.4a
% RDA or PRI contribution^c^ (male/female)	Ca	FAO	1000/1000	0.1/0.1	0.2/0.2	0.3/0.3	0.3/0.3	0.3/0.3	0.4/0.4
		EFSA	950/950	0.1/0.1	0.2/0.2	0.3/0.3	0.3/0.3	0.3/0.3	0.4/0.4
	Fe	FAO_5_	27.4/58.8	0.7/0.3	0.9/0.4	1.0/0.5	1.6/0.7	1.2/0.6	1.2/0.6
		FAO_10_	13.7/29.4	1.4/0.7	1.7/0.8	2.0/1.0	3.1/1.5	2.5/1.2	2.5/1.1
		FAO_12_	11.4/24.5	1.7/0.8	2.0/1.0	2.5/1.1	3.8/1.8	3.0/1.4	3.0/1.4
		FAO_15_	9.1/19.6	2.1/1.0	2.6/1.2	3.1/1.4	4.7/2.2	3.7/1.7	3.7/1.7
		EFSA	11/16	1.8/1.2	2.1/1.5	2.5/1.8	3.9/2.7	3.1/2.1	3.1/2.1
	Zn	FAO_high_	4.2/3	3.3/4.6	3.4/4.7	3.5/4.8	3.4/4.8	2.5/3.6	2.4/3.3
		FAO_moderate_	7/4.9	2.0/2.8	2.0/2.9	2.1/3.0	2.0/2.9	1.5/2.2	1.4/2.0
		FAO_low_	14/9.8	1.0/1.4	1.0/1.4	1.0/1.5	1.0/1.5	0.8/1.1	0.7/1.0
		EFSA_300_	9.4/7.5	1.5/1.8	1.5/1.9	1.5/1.9	1.5/1.9	1.1/1.4	1.1/1.3
		EFSA_600_	11.7/9.3	1.2/1.5	1.2/1.5	1.2/1.6	1.2/1.5	0.9/1.1	0.9/1.1
		EFSA_900_	14.0/11.0	1.0/1.2	1.0/1.3	1.0/1.3	1.0/1.3	0.8/1.0	0.7/0.9
		EFSA_1200_	16.3/12.7	0.8/1.1	0.9/1.1	0.9/1.1	0.9/1.1	0.7/0.8	0.6/0.8
Free Phosphorus^b^,mg/100 g			–	91 ± 2d	97 ± 2e	92 ± 0d	64 ± 4a	83 ± 1c	72 ± 0b
Ins*P*_*6*_^b^, g/100 g			–	1.14 ± 0.04e	1.24 ± 0.02f	0.93 ± 0.05d	0.61 ± 0.01c	0.51 ± 0.02b	0.13 ± 0.00a
Ins*P*_6_/Ca^d^ ˃ 0.24, mol/mol			–	2.23	1.60	0.69	0.52	0.54	0.10
Ins*P*_6_/Fe^d^ ˃ 1.0, mol/mol			–	22.2	20.2	12.5	5.4	5.9	1.6
Ins*P*_6_/Zn^d^ ˃ 15.0, mol/mol			–	36.7	38.9	28.4	19.3	22.0	6.2

^a^Values are expressed as mean ± standard deviation (*n* ≥ 3). Identical letters within a row indicate no significant differences at the 95% confidence level. ^b^Dry matter. ^c^Percentage contribution to FAO of RDAs (Recommended Daily Allowances) and EFSA of PRIs (Population Reference Intakes) based on a daily intake of 5 g of quinoa fibre, assuming no mineral absorption inhibitors. RDAs/PRIs in mg/day for adults (male/female) ≥ 18 years [21–24]. FAO iron RDAs are adjusted for dietary iron bioavailability: FAO_X_ (X: 5, 10, 12, or 15%) [21]; FAO zinc bioavailability is classified by dietary phytate content: FAO_high_ (Ins*P*_6_/mineral < 5), FAO_moderate_ (5–15), and FAO_low_ (> 15) [21]. EFSA zinc PRIs vary with daily Ins*P*_6_ intake: EFSA_300_, EFSA_600_, EFSA_900_, EFSA_1200_ (mg/day) [24]; ^d^threshold Ins*P*_6_/mineral ratios associated with reduced mineral bioavailability in humans: Ca, Fe or Zn [25–27]. Abbreviations: FAO: Food and Agriculture Organization of the United Nations, EFSA: European Food Safety Authority; Ins*P*_6_: *myo*-inositol hexakisphosphate or phytate

Mineral contribution percentages were estimated for an intake of 5 g for each quinoa fibre or its whole flour, relative to adult Recommended Daily Allowances (RDAs) from FAO [21] and Population Reference Intakes (PRIs) by EFSA [22–24], assuming no mineral absorption inhibitors (Table 2). Iron requirements vary with dietary bioavailability: around 15% in Western diets, but lower in women (10–12%) and in vegetarian or vegan diets (5%) due to the presence of inhibitors from cereals, legumes, and vegetables [21]. For zinc, FAO categorises bioavailability based on the phytate-to-mineral ratio (Ins*P*_6_/mineral: <5, 5–15, > 15 for high, moderate, and low), while EFSA defines four levels based on daily Ins*P*_6_ intake (300–1200 mg) [24]. An increase in the contribution of calcium to the RDAs/PRIs was observed in quinoa fibres compared to their corresponding whole flours, with white quinoa fibre exhibiting a threefold increase. Iron contribution also increased, doubling in the case of white quinoa fibre. Although calcium and iron levels were enriched in the fibre fractions, zinc content decreased notably in red and black quinoa fibres. Consequently, the overall contribution of zinc to the RDAs/PRIs remained comparable to, or slightly lower than, that of the original flours.

### Phytates and Estimated Mineral Bioavailability

The values for phosphorus, phytates, and phytate-mineral molar ratios are presented in Table 2. Free phosphorus was significantly higher in flour samples than in their corresponding fibre fractions. While part of it may have been removed during wet milling due to solubilisation, its presence in the fibre fractions could be related to the action of endogenous phytase during drying at 40 °C, close to its optimal activity in cereals [30]. The phytate content of white, red and black quinoa fell within the range reported in previous studies (0.61–1.34 g/100 g) [16], with red quinoa exhibiting the highest level. In all cases, the isolated fibre fractions showed markedly reduced phytate levels compared to their respective whole grains. This reduction is primarily attributed to the steeping stage of the wet milling process, during which phytic acid can leach into the steeping water, thereby decreasing its concentration in the retained solid fractions. Moreover, the steeping conditions (notably the hydration time and temperature) may facilitate the partial activation of endogenous phytases naturally presents in quinoa. These enzymes hydrolyse phytic acid into lower inositol phosphates, which are more soluble and exert less inhibitory effect on mineral absorption. Phytate reductions of 46, 59 and 85% were observed in the fibres derived from white, red, and black quinoa, respectively, compared to the phytate content of the corresponding whole grains. As a result, all fibre samples demonstrated improved molar ratios of phytate to minerals, suggesting enhanced potential for mineral bioavailability. In particular, black quinoa fibre showed the most favourable profile, with phytate-to-calcium and phytate-to-zinc falling below critical thresholds for mineral absorption inhibition (Table 2). These findings highlight a clear nutritional advantage of quinoa fibres ingredients obtained via wet milling, as the process not only concentrates dietary fibre but also lowers phytate content. This contrasts with dry milling techniques, where phytate levels remain largely unchanged, potentially limiting bioavailability.

### Physical Properties of Quinoa Fibres

**Fig. 2:**
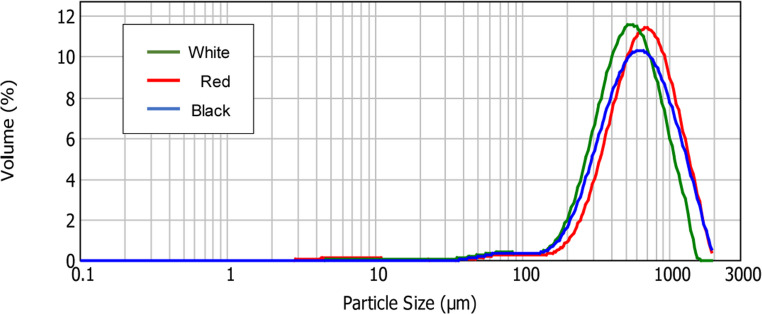
Particle size distribution of quinoa fibres

Particle size distribution values for quinoa fibre samples are shown in Table 3. Particle size is a key parameter influencing both the functional properties of dietary fibre in the human body and the quality of fibre-enriched food products [31]. It directly affects characteristics such as texture, appearance, digestibility and viscosity, which in turn modulate physiological responses such as glycaemic index [32]. All quinoa fibre samples showed unimodal distributions (Fig. 2), with significant differences observed among varieties (*p* < 0.05) (Table 3). Red quinoa fibre exhibited the largest median particle size (D50 parameter). In terms of dispersity (Đ), black quinoa fibre displayed significantly higher values, suggesting a larger particle size distribution, likely due to small fibre aggregates formed during wet milling, as a result of its anisotropic cell wall structure and high-water affinity. No significant differences in dispersity were found between white and red quinoa fibres. Differences in particle size can have nutritional implications. Fibres fractions with smaller particle sizes, such as those observed in white and black quinoa fibres, tend to exhibit higher surface area, which may enhance glucose adsorption, delay enzymatic hydrolysis of starch, and thereby contribute to improved glycaemic control [33]. Moreover, a finer particle size would increase the number of particles per unit weight, improving dispersibility and functional integration in food matrices [34]. Therefore, the quinoa fibres with smaller particles sizes may offer functional and nutritional advantages when incorporated into low-GI formulations or functional foods targeting glycaemic modulation.

**Table 3:** Techno functional properties of Quinoa fibre fractions

Parameter	Units	Quinoa fibres		
		White	Red	Black
Particle size distribution				
D [4.3]	µm	527 ± 9a	716 ± 47b	677 ± 50c
D10	µm	255 ± 1a	314 ± 32b	278 ± 25a
D50	µm	525 ± 11a	656 ± 40c	603 ± 46b
D90	µm	973 ± 19a	1223 ± 68c	1199 ± 85b
Đ	µm	1.37 ± 0.01a	1.39 ± 0.03a	1.53 ± 0.03b
Techno functional properties				
WHC	g water/g sample	6.3 ± 0.4a	10.8 ± 0.1b	6.0 ± 0.3a
OHC	g oil/g sample	4.7 ± 0.1a	7.1 ± 0.2b	4.2 ± 0.5a
SC	mL/g sample	1.1 ± 0.2a	3.8 ± 0.3c	2.0 ± 0.5b

Values are expressed as mean ± SD (*n* = 3). SD values followed by same letter in the same line are not significantly different at 95% of confidence. Abbreviations: D [4.3] (Medium particle diameter); D10 (smaller particle diameter; 10% of the particles are smaller than the indicated particle size); D50 (mean diameter, 50% of the particles are smaller than the indicated particle size); D90 (larger particle diameter; 90% of the particles are smaller than the indicated particle size); Đ (dispersion); WHC (water holding capacity); OHC (oil holding capacity), and SC (swelling capacity)

**Fig. 3:**
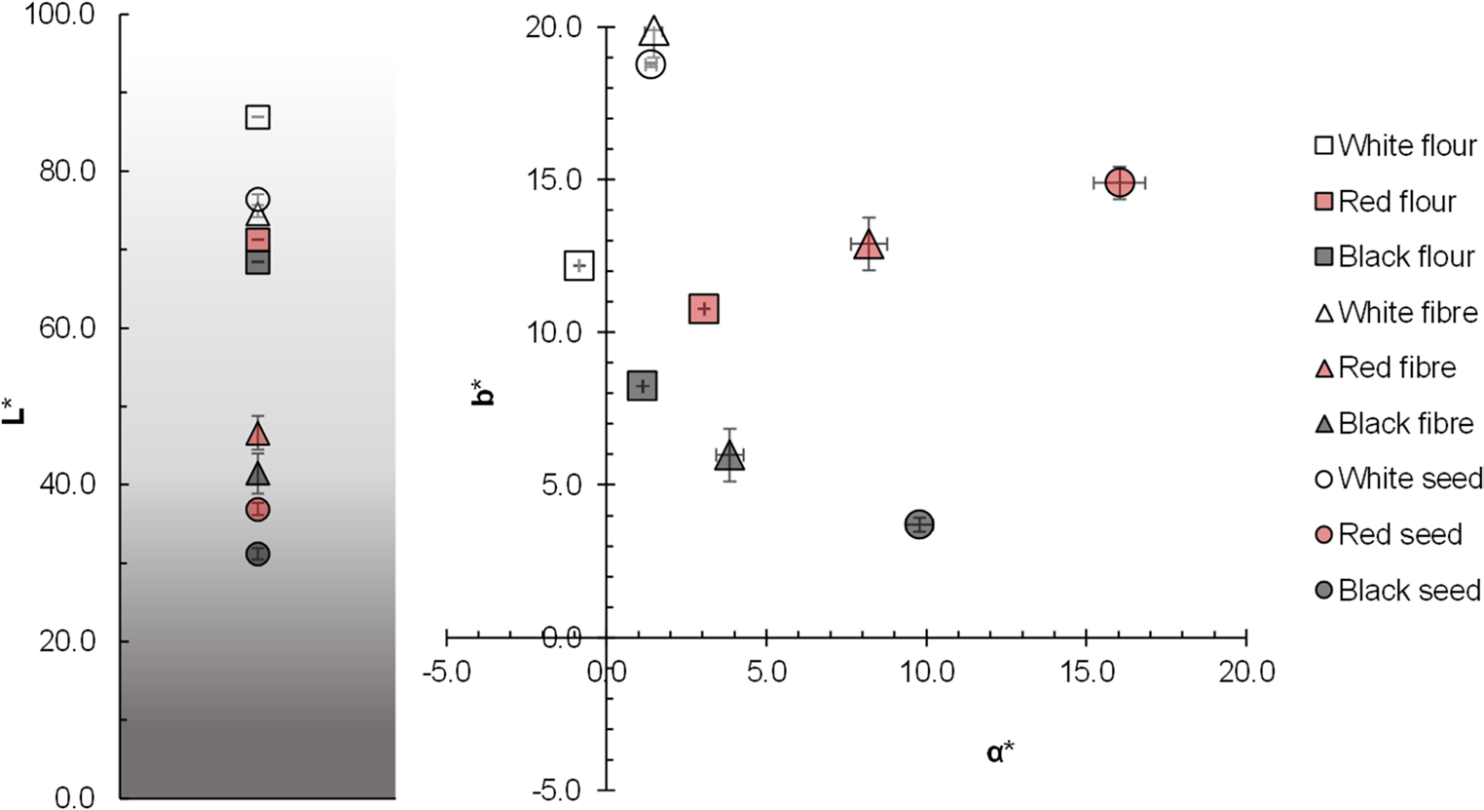
Colour parameters of quinoa seeds, whole flours and fibres

**Fig. 4:**
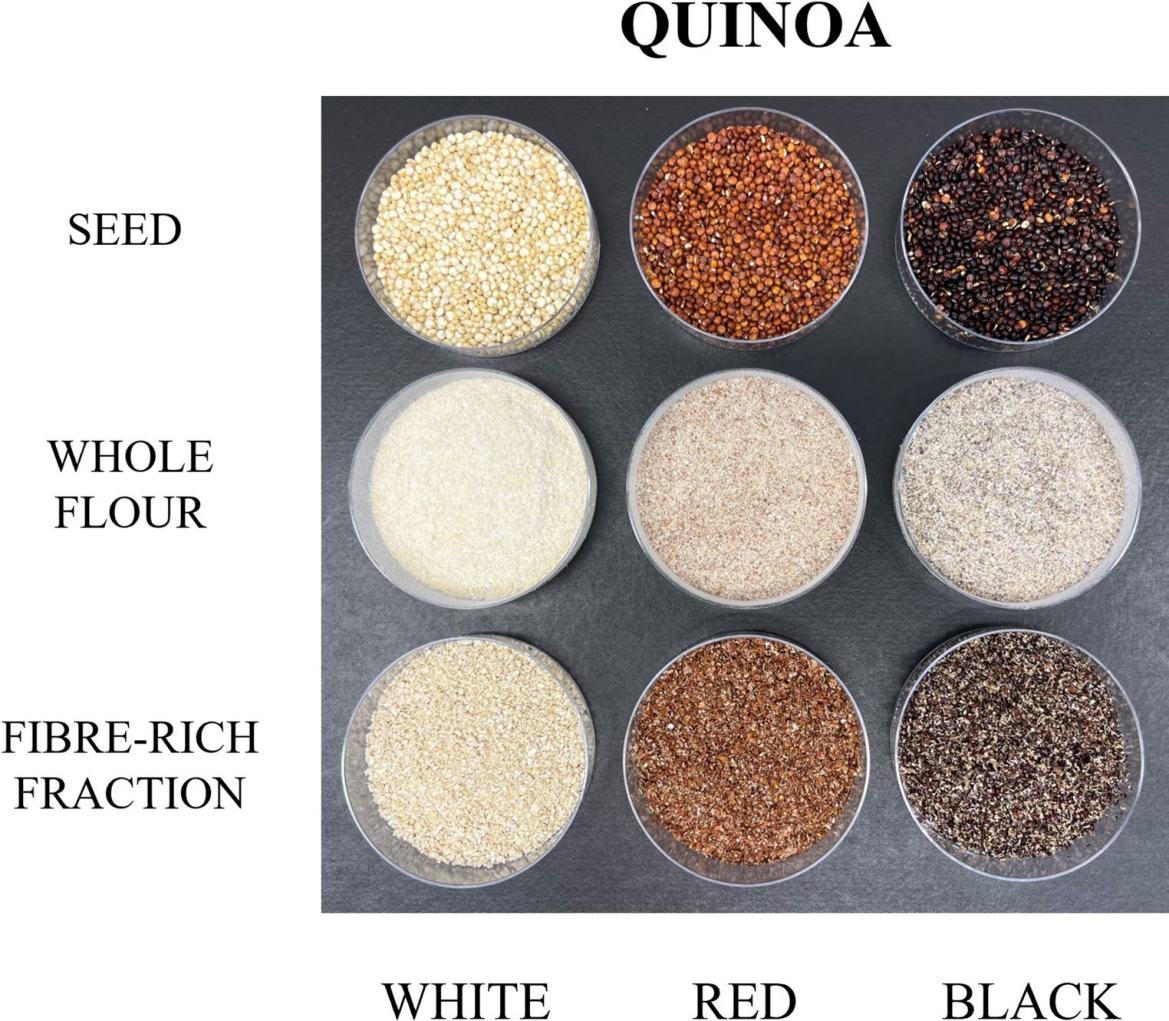
Visual aspect of quinoa seeds, whole flours and fibres

The colour of the different dietary fibre sources is influenced by various factors, such as the variety of the raw material and the processing methods used to obtain the fibre fractions [35]. The total colour differences (∆E) between quinoa fibres and their original grains were significantly smaller than those observed between the corresponding flours and grains (Table S1, Supplementary Material). This finding indicates that the fibre fractions are particularly enriched in pigments and compounds located in the outer grain layers. Figure 3 shows the colour parameters of fibres, whole flours and quinoa grains, while its visual appearance is reflected in Fig. 4. Regarding the lightness parameter (L*), white quinoa fibre exhibits the highest value, appearing closer to white in comparison with red and black quinoa fibres. In terms of the a* parameter, all fibre samples showed a tendency towards red, with red quinoa fibre displaying the most pronounced red hue, followed by black quinoa fibre. Analysis of the b* values indicates that all fibre types possess a yellow component, with white quinoa fibre being the most yellowish and black quinoa fibre the least. Compared to their respective fibre fractions, whole quinoa flours exhibited higher lightness across all varieties. These colour differences arise because whole flours contain more of the starchy endosperm whereas fibre fractions retain higher concentrations of pigmented compounds located in the outer grain layers, such as phenolic compounds, flavonoids, and betalain pigments, particularly in red and black quinoa varieties [36]. For instance, Tang et al. [37] detected significant levels of phenolics and betanin (a red betacyanin) in red and black quinoa seeds. Betacyanins and betaxanthins are largely absent in white quinoa, which explains the more neutral, yellowish tint of its fibre fractions, compared to the darker hues of pigmented varieties. The presence of these pigments not only depends the colour but can also offer functional benefits: phenolic compounds and betalains are known antioxidants with potential health-promoting properties. Ballester et al. [2] reported that darker fibre quinoa varieties (5% inclusion level) enhanced the colour and total phenolic content of bakery products (21.06 mg gallic acid equivalent/g d.m. compared to 14.49 mg gallic acid equivalent/g d.m. in control bread). Therefore, colour differences among quinoa fibre types are not merely visual but reflect differences in phytochemical composition. This offers opportunities to tailor ingredient selection based on desired visual and functional attributes in food product development. The more pigmented quinoa fibres could be suitable for applications where a darker or more rustic tone is required. For instance, red or black quinoa fibres may be used in high-fibre granolas, cereal bars, cookies, meat analogues or dark-coloured bakery products. Additionally, they could contribute to the visual appeal of artisanal or health-positioned foods, where natural variation and depth of colour are often valued by consumers.

### Techno-Funcional Properties

Among the samples studied, red quinoa fibre showed the highest water-holding capacity (WHC), significantly exceeding that of white and black quinoa (Table 3). This difference was primarily attributed to particle size, with finer particles retaining less water. These findings align with previous studies indicating that milling decreases WHC by disrupting the fibre matrix and facilitating water loss [5]. Red quinoa fibre, despite having a higher proportion of insoluble fibre, retained more water than white quinoa fibre, which contains more soluble fibre. While soluble fibre is generally associated with higher WHC and the formation of viscous solutions [6], this was not reflected in the results, suggesting that physical structure had a greater influence than insoluble-to-soluble fibre ratio in this case. Moreover, the lower starch content in red quinoa fibre may have enhanced WHC, as ungelatinized starch exhibits minimal swelling at room temperature and may hinder water retention. Differences in extraction yield could also contribute to these results, as higher yields may include more non-fibrous components, such as residual starch, which could reduce the water-binding capacity in the fibre matrix. Notably, red quinoa fibre reached WHC levels comparable to fruit and vegetables fibres (> 7 g water/g sample), while white and black fibres were closer to cereal brans (3–5 g water/g sample) [18].

Oil-holding capacity (OHC) is primarily influenced by the porosity of the fibre, rather than by its chemical affinity for lipids, and can be reduced by processing steps such as milling and washing, which decrease particle size and limit pore availability [8]. Among the fibres analysed (Table 3), red quinoa fibre presented the highest OHC, showing statistically significant differences (*p* < 0.05) compared to white and black quinoa fibre. This result was likely associated with its larger particle size, which facilitated greater oil retention within the fibre matrix. The elevated OHC of red quinoa fibre, combined with its high-water retention capacity, highlights its potential for reducing energy density and enhancing satiety in food formulations. Compared with reference values (1.5–7 g oil/g sample) [18], quinoa fibres, particularly red, showed strong oil-binding potential, while white and black also performed above many conventional cereal fibres.

Swelling capacity (SC) is influenced by the ratio of soluble to insoluble fibre, particle size, and porosity. Fibres with higher SC can improve product freshness and stability by preventing volume loss, while also promoting satiety and increased faecal bulk following ingestion [9]. As shown in Table 3, red quinoa fibre exhibited the highest SC, with significant differences compared to white and black quinoa fibres. This behaviour may be associated with its larger particle size, which could favour greater water uptake and volumetric expansion. Overall, the swelling capacity values of all quinoa fibres were lower than those generally reported for fruit-derived fibres, yet they remained within the range commonly described for cereal-based fractions [18].

In this context, the techno-functional profile of quinoa fibres bridges the gap between cereal and fruit fibres: red quinoa variety resembles fruit fibres with high hydration and textural capacities, while white and black fibres behave more like cereal brans. This duality highlights quinoa fibre as a versatile ingredient with both nutritional and technological relevance.

## Conclusions

Isolated fibres from different quinoa varieties exhibit promising nutritional and techno-functional properties, supporting their potential application in the food industry. Among them, black quinoa fibre stands out for its high total and insoluble fibre content, and the highest extraction yield (20.6% d.m.), making it a valuable ingredient to enhance daily dietary fibre intake. In fact, a 5 g serving of this fraction could provide up to 14% of the recommended daily intake, an amount that would require consuming 35 g of the original flour to achive the same contribution. Notably, black quinoa fibre also showed a phytate-to-calcium and phytate-to-zinc molar rations falling below critical thresholds for mineral absorption inhibition, suggesting improved bioavailability of key micronutrients such as calcium and zinc. Red quinoa fibre, while yielding slightly lower recovery, may offer additional technological advantages, including enhanced water- and oil-holding capacities and higher swelling capacity, which could contribute to improve texture and stability in food formulations. These properties make it particularly suitable for bakery, meat analogues, or high-fibre snack applications. The predominance of insoluble fibre across all fractions could support potential physiological benefits, such as enhanced satiety, improved gut motility, and reduced fat and carbohydrate absorption, reinforce their value functional food ingredients. Moreover, quinoa fibres isolated through wet milling processes tend to exhibit lower phytic acid content. This may favour mineral bioavailability in food products formulated with these fibres, even in non-fermented applications. In addition, calcium and iron concentrations were concentrated in the fibre fractions compared to the whole quinoa flours, further enhancing their nutritional value. Nonetheless, certain limitations should be acknowledged. The relatively low content of soluble fibre may restrict the potential for targeting health benefits related to blood glucose or cholesterol modulation. Additionally, the functionality of these fibres in real food matrices and their sensory impact remain to be fully assessed. Future research should focus on evaluating the in vivo nutritional benefits, including mineral bioavailability and possible prebiotic effects, as well as exploring synergies with other fibre sources or processing strategies to optimise their applicability in a wider range of food products. In brief, wet-milled quinoa fibres, particularly those from black and red varieties, represent a highly promising ingredient for the formulation of nutritionally enhanced and functionally enriched foods, combining high fibre content, improved mineral bioavailability, and desirable techno-functional properties.

## Supplementary Information

Below is the link to the electronic supplementary material.

ESM 1 (DOCX 50.1 KB)

## Data Availability

No datasets were generated or analysed during the current study.
