# Supplementary material for: Quinoa Fibre Isolated By Wet Milling as a New Ingredient for Food Enrichment: Nutritional Value and Technological Properties
Source: Plant Foods Hum Nutr. 2025 Oct 18;80(4):175. doi: 10.1007/s11130-025-01409-5 (PMC12535488; doi:10.1007/s11130-025-01409-5)
Supplement: Supplementary file 1 — (DOCX 50.1 KB) [file 11130_2025_1409_MOESM1_ESM.docx]

**QUINOA FIBRE ISOLATED BY WET MILLING AS A NEW INGREDIENT FOR FOOD ENRICHMENT: NUTRITIONAL VALUE AND TECHNOLOGICAL PROPERTIES**

Alonso-Álvarez, A., Haros, C.M.*

Cereal Group, Instituto de Agroquímica y Tecnología de Alimentos (IATA-CSIC), Valencia, Spain

*Corresponding author. Mailing address: Instituto de Agroquímica y Tecnología de Alimentos (IATA-CSIC), Av. Agustín Escardino 7, Parque Científico, 46980 Paterna, Valencia, Spain. Tel: +34 96 390 00 22; E-mail: mharos@iata.csic.es

<https://orcid.org/0000-0001-7904-0109>

**Supplementary material**

**Material and Methods**

**Chemical proximal composition**

Moisture and starch contents in quinoa flours and fibres were assessed in accordance with AOAC Methods 925.09 and 996.11 [1]. Lipid content was determined via the Randall extraction procedure, employing diethyl ether solvent (AOAC Methods 2003.05 and 2003.06 [2] using a SER 158/3 Solvent Auto Extractor (Velp Scientifica, Usmate, Italy). Protein concentration was quantified using the Dumas combustion method (Nx6.25), as outlined in ISO/TS 16634-2 [3]. Ash content was measured following the standard AACC Method 08-03 [4]. Soluble and insoluble dietary fibre were determined by an enzymatic-gravimetric method (AOAC Method 991.43 [1]); total dietary fibre was calculated as the sum of these two fractions. All measurements were conducted in triplicate.

**Determination of phytate**

Phytic acid (Ins*P*_6_) content in whole quinoa flours and fibre samples was determined in triplicate using a commercial kit (K-Phyt 07/11, Megazyme, Ireland) and a spectrophotometer (SPECTROstar Nano, BMG LabTech, Germany). Phytates were extracted from 0.5 g of sample with 10 mL HCl and hydrolysed enzymatically with phytase and alkaline phosphatase. Inorganic phosphate released was quantified colourimetrically according to AOAC Method 986.11 [5] using ammonium molybdate and ascorbic acid. Absorbance was measured at 655 nm and phosphate concentration was calculated from a standard phosphorus calibration curve.

**Determination of minerals**

Samples of 0.25 grams from each whole quinoa flour and fibre were digested with 14 M nitric acid (Merck, Germany) and hydrogen peroxide (Panreac Química, Spain) in perfluoroalkoxy alkane vessels. The digestion was carried out using a microwave-assisted reaction system (One Touch Technology MARS6, CEM, Vertex, Spain). Calcium, iron and zinc concentrations were determined by inductively coupled plasma mass spectrometry (iCAP RQ ICP-MS) from ThermoFisher Scientific (Waltham, MA, USA) [6]. The analyses were performed in hexaplicate.

**Physical and techno functional properties of fibre fractions**

The particle size distribution of the dietary fibre fraction was determined using the MasterSizer 2000 laser diffraction equipment (Malvern Instruments Ltd., UK), equipped with a Scirocco 2000 dispersing unit for dry samples, following ISO 13320 guidelines [7]. Measurements were processed with MasterSizer 2000 software v.5.60. Applying Mie scattering theory, results were expressed as the volume percentage of particles across diameter ranges (µm). The distribution was characterised by the volume mean diameter (D[4,3]) – a volume-weighted mean particle size calculated as the ratio of the fourth to the third moment of the distribution - and the percentiles d(0.1), d(0.5) and d(0.9), which represent the diameters below which 10 %, 50 % and 90 % of the total particle volume is contained. Water and oil holding capacity (WHC and OHC, respectively) were assessed according to the method outlined by Robertson et al. [8], while swelling capacity (SC) was carried out following the method described by Gómez-Ordóñez et al. [9].

The colour attributes of quinoa grains, wholemeal flours, and derived fibres were evaluated using a Chroma Meter CR 400 (Konica Minolta Sensing Inc., Tokyo, Japan), previously standardised against a white calibration plate (Y = 84,1, x = 0.3151, y = 0.3210). The CIELAB coordinates: lightness (L*), red-green axis (a*), and yellow-blue axis (b*); were measured. Each sample was analysed in triplicate.

**Statistical Analysis**

Statistical differences among the samples were evaluated using one-way ANOVA followed by Fisher’s Least Significant Differences (LSD) test. Analyses were performed with Statgraphics Plus version 7.1 (Bitstream, Cambridge, MN, USA), considering *p* < 0.05 as the threshold for significance.

**References**

1. Association of Official Analytical Chemists (AOAC) (1996) Official Methods of Analysis. AOAC International. Methods 925.09: solids (total) and moisture in flour, Method 945.16: oil in cereal adjuncts, Method 991.43: total, soluble, and insoluble dietary fiber in foods. Method 996.11: starch (total) in cereal products, Amyloglucosidase/α-Amylase, in Official Methods of Analysis. 15th edn. Arlington, VA, USA.

2. Association of Official Analytical Chemists (AOAC) (2005) Official Methods of Analysis. AOAC International. Method 2003.05: Crude Fat in Feeds, Cereal Grains, and Forages - Randall/Soxhlet/Diethyl Ether Extraction-Submersion Method. 18th Ed. Gaithersburg, MD.

3. International Organization for Standardization (ISO) (2016) Food Products-Determination of the Total Nitrogen Content by Combustion According to the Dumas Principle and Calculation of the Crude Protein Content—Part 1 and 2: Cereals, Pulses and Milled Cereal Products (ISO/TS16634-1 and ISO/TS16634-2). Geneva, Switzerland.

4. Association of Analytical Cereal Chemists (AACC) (1995) AACC Approved Methods of Analysis. Method 08-03: Total ash, Method 46-13: crude protein—Micro-Kjeldahl Method, in approved methods of American Association of Cereal Chemistry. 11th Ed. Saint Paul, Minnesota, USA.

5. Association of Official Analytical Chemists (AOAC) (1990) Official Methods of Analysis. AOAC International. Method 986.11: Phytate in foods. Anion Exchange Method. 15th Ed. Arlington, VA, USA.

6. Sánchez A, Vélez D, Devesa V (2024) Processes influencing the toxicity of microplastics ingested through the diet. Food Chemistry 456:139947 https://doi.org/10.1016/j.foodchem.2024.139947

7. International Organization for Standardization (ISO) (2020) Particle size analysis-Laser diffraction methods (ISO 13320:2020). Geneva, Switzerland.

8. Robertson JA, de Monredon FD, Dysseler P, Guillon F, Amado R, Thibault J-F (2000) Hydration Properties of Dietary Fibre and Resistant Starch: a European Collaborative Study. LWT - Food Science and Technology 33(2):72–9 https://doi.org/10.1006/fstl.1999.0595

9. Gómez-Ordóñez E, Jiménez-Escrig A, Rupérez P (2010) Dietary fibre and physicochemical properties of several edible seaweeds from the northwestern Spanish coast. Food Research International 43(9):2289-2294 https://doi.org/10.1016/j.foodres.2010.08.005

**Table 1S.** Colour differences in quinoa grains, flours and fibre-rich fractions

| **Parameter** | Control | Quinoa Flours | | | Quinoa Fibres | | |
| --- | --- | --- | --- | --- | --- | --- | --- |
|  |  | White | Red | Black | White | Red | Black |
| *ΔE*rw* | 6.80±0.02a | 11.6±0.0b | 23.8±0.0b | 25.6±0.0b | 25.6±0.7b | 48.6±2.0c | 52.2±2.5c |
| *ΔE*c* | - | 5.09±0.02a | 19.9±0.0b | 22.3±0.0c | 19.7±0.6b | 45.1±2.1d | 49.5±2.6d |
| *ΔE*g* | - | 12.6±0.1b | 37.0±0.0c | 38.5±0.0c | 2.2±0.1a | 12.6±0.3b | 12.1±0.2b |

ΔE*= $\sqrt{(\Delta L*)^{2}+ (\Delta a*)^{2}+(\Delta b*)^{2}.}$ *ΔE*rw (Total colour difference between ingredient and the reference white; rw parameters: L* =93.4; a* = 0.2, and b* = 2.6); ΔE*c (Total colour difference between ingredient and the control, wheat flour whose parameters are: L* = 90.6; a* = -0.97, and b* = 8.7); ΔE*g (Total colour difference between ingredient and the original grain).*
